# Home Dermoscopy During the COVID-19 Pandemic

**DOI:** 10.5826/dpc.1004a91

**Published:** 2020-10-26

**Authors:** Andreas Blum, Michelle Menzies

**Affiliations:** 1 Public, Private and Teaching Practice of Dermatology, Konstanz, Germany; 2 Skintography, Sydney, Australia

**Keywords:** home dermoscopy, pandemic, COVID-19

With the sudden onset of the COVID-19 pandemic crippling the world, dermatologists are using teledermatology to care for their patients remotely. Diagnosing skin cancer with only clinical photos alone is difficult, and as patients do not have access to a dermatoscope and are isolated at home, a simple technique using a disinfection spray, cooking oil, or water and a camera has shown promising results. The patient applies the immersion fluid on the lesion of concern and takes 1–3 focused images with a camera distance of ~20–30 cm using ambient indoor or outdoor light for illumination (Figure 1). To obtain better image quality, hairs should be removed and air bubbles should be avoided. The diagnosing dermatologist will enlarge the focused images on the computer screen, analyze the visible morphologic features and colors of the upper layers of the epidermis, and make further recommendations.

Two cases demonstrate this simple technique:

1. A 20-year-old male with a new nodular lesion on his shoulder (Figure 2). The enlarged image showed dotted vessels (arrows) and pink color. Urgent surgery with histopathology was recommended to exclude the spitzoid melanoma or to confirm the benign Spitz nevus (as in this case).
2. A 24-year-old female with a growing lesion on her leg (Figure 3). The enlarged image revealed eccentric streaks (arrows) and an eccentric hyperpigmentation (asterisk) with dark brown and black colors, and histopathology confirmed the diagnosis of an early invasive melanoma (tumor thickness 0.4 mm).

It must be mentioned that this simple approach of “home dermoscopy” does not replace medical examination with a real dermatoscope.

## Figures and Tables

**Figure 1:**
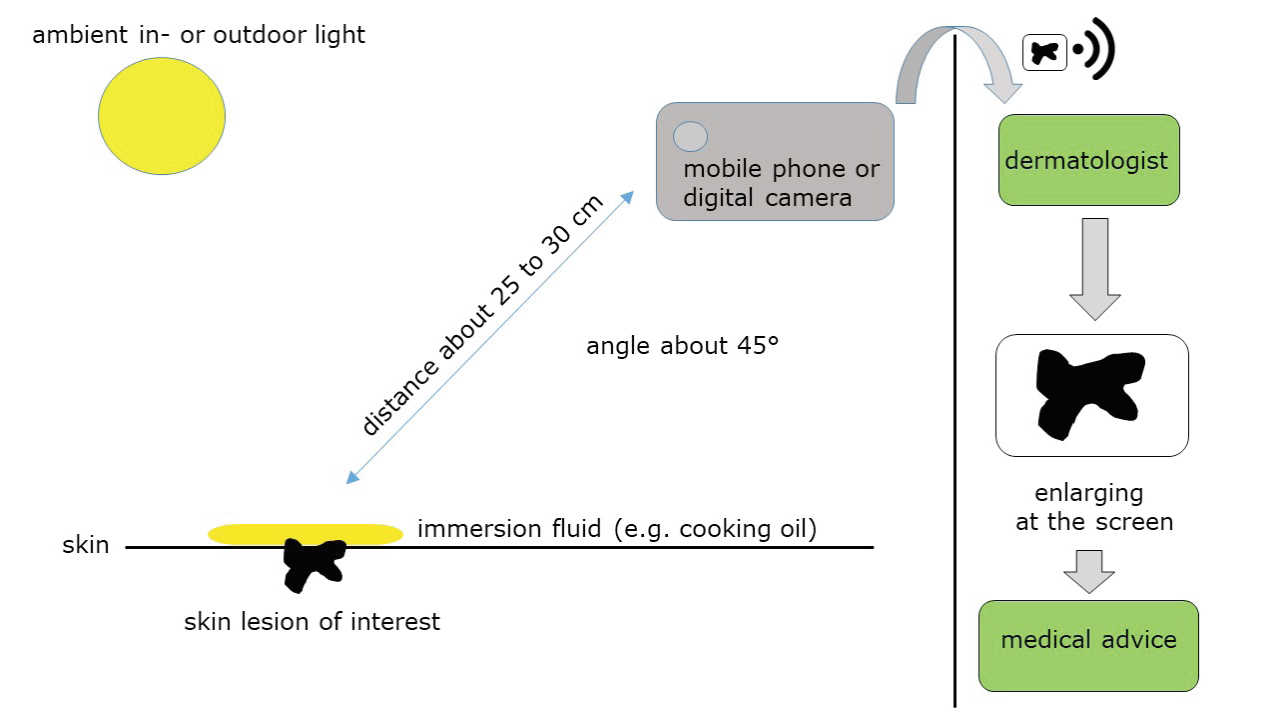
Dermatoscope-less recording of a skin lesion of interest with visible morphologic features.

**Figure 2:**
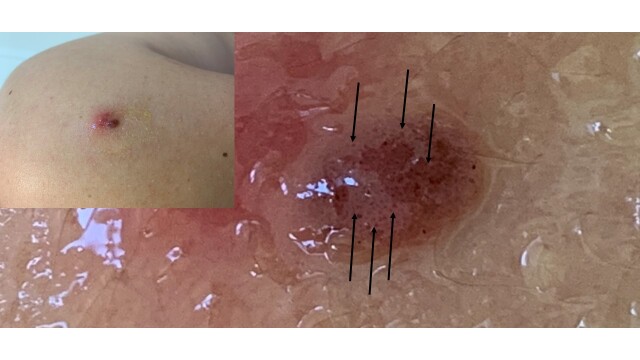
Enlarged image on screen revealed morphologic features of dotted vessels (arrows) and pink color; histologically a benign Spitz nevus was confirmed.

**Figure 3:**
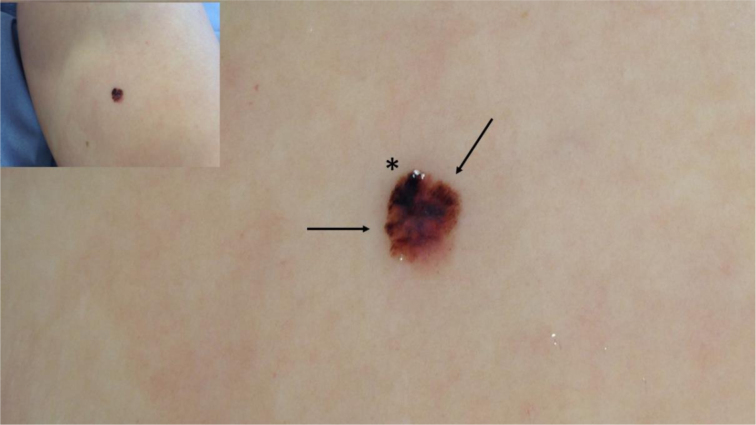
Enlarged image on screen revealed morphologic features of eccentric streaks (arrows) and an eccentric hyperpigmentation (*) with dark brown and black colors; histologically an early invasive melanoma (tumor thickness 0.4 mm) was confirmed.

